# Does laughing have a stress-buffering effect in daily life? An intensive longitudinal study

**DOI:** 10.1371/journal.pone.0235851

**Published:** 2020-07-09

**Authors:** Thea Zander-Schellenberg, Isabella Mutschler Collins, Marcel Miché, Camille Guttmann, Roselind Lieb, Karina Wahl

**Affiliations:** Division of Clinical Psychology and Epidemiology, Department of Psychology, University of Basel, Basel, Switzerland; Hunter College, UNITED STATES

## Abstract

Positive affect is associated with alleviating mental and physiological stress responses. As laughter is a common physiological operationalization of positive affect, we investigated whether the effects of experiencing a stressful event on stress symptoms is lessened by frequency and intensity of daily laughter. Using an intensive longitudinal design, we ambulatory assessed the self-reported experience of stressful events, stress symptoms and the frequency as well as the intensity of laughter in university students’ daily lives. Our hierarchical ecological momentary assessment data were analyzed with multilevel models. The results support the stress-buffering model of positive affect: We found that the frequency of laughter attenuated the association between stressful events and subsequent stress symptoms. The level of intensity of laughter, however, was found to have no significant effect. Future studies should use additional psychophysiological indicators of stress and straighten out the differential contributions of frequency and intensity of daily laughter.

## Introduction

Positive affect is understood as a state of experiencing pleasure. For example, people feel happy, joyful, calm, enthusiastic, excited, and/or satisfied when being in a positive affect state [1, 2]. Using various forms of operationalization, several studies report associations of positive affect with a wealth of positive outcomes. For example, positive affect is positively associated with meaning in life [3], future life satisfaction [4], positive health behaviors [5], restful sleep [6], and longevity [7]; for reviews, see [8, 9]; and [10]; see [5] as well as [11] for more nuanced approaches.

Moreover, studies observed that positive affect is not only associated with positive outcomes but also plays a crucial role in alleviating stress. Stress can negatively impact physical and mental health. It has been linked to various medical conditions such as fatigue, back pain, and cardiac and immunosuppressive conditions [12, 13] and increases the risk of developing anxiety and depression (e.g., [14]). Stress-buffering models of positive affect suggest that positive affect has the potential to reduce these negative health-harming consequences of psychological stress on mind and body. In particular, it has been proposed that positive affect attenuates negative consequences of stress by reducing the physiological stress reactivity or by altering coping strategies of the stressed individual [8].

There is a substantial amount of empirical evidence providing support for the assumptions of the stress-buffering model of positive affect. Bolstering the moderating effect of positive affect, study results suggest an “undoing effect of positive emotions” on the “cardiovascular aftereffects [*sic*] of negative emotions” [15]. Fredrickson [1], for example, showed healthy participants fear-evoking movie sequences and measured their cardiac responses. Afterwards, participants either watched a sad, neutral, or amusing movie. The cardiac responses of those participants who watched the amusing movie recovered faster to baseline level than the cardiac responses of the other two groups, supporting the assumptions of the stress-buffering model of positive affect (for further examples, see also Kraft and Pressman [16]). A recent prospective study showed that the stress-buffering function of positive affect is more pronounced in individuals who experience high levels of stress [17]. Along these lines, a recent review of investigations into humor and pain reported a humor-triggered increased pain tolerance for experimental pain and a keen sense of humor as a coping mechanism for chronic pain conditions [18].

Laughter can be seen as the physiological expression of positive affect and, thus, positive affect has often been operationalized as frequency or intensity of laughter (or smiling). It is assumed that people laugh depending on the level of pleasure experienced. If the level of pleasure experienced is high, a laugh follows; where it is lower, people rarely exhibit a smile [19]. Martin and Kuiper [20] estimate that individuals typically laugh 18 times per day, mostly in interaction with others. They also report daytime and gender differences in the frequency of daily laughter. A meta-analysis revealed that, on average, women smile more than men [21]. More recent studies also report differences in the frequency of smiling between cultures [22].

The link between laughter and a reduced stress response has mostly been investigated in the laboratory (e.g., [15, 16]) or in prospective studies using typically two measurement points (e.g., [17]). Whether the relationship between stress and positive affect also applies to daily life experiences when stress and positive affect might happen in close temporal approximation has not been investigated so far. Thus, the current study investigated the stress-buffering model of positive affectivity using an intensive prospective longitudinal study design in a real-life setting with high ecological validity. In particular, the aim of the study was to examine in a student sample whether the effect of experiencing a stressful event on stress symptoms is attenuated by frequency and intensity of laughter. Based on the empirical evidence summarized above, we derived the following hypotheses: Frequency of laughter attenuates the association between prior experience of stressful events and subsequent experience of stress symptoms in daily life (hypothesis 1); intensity of laughter attenuates the association between prior experience of stressful events and subsequent experience of stress symptoms in daily life (hypothesis 2).

## Methods

### Design

Our study, which was conducted over a period of three months from March to May 2018, is based on an intensive prospective longitudinal study design using the Experience Sampling Method (ESM; [23]). Data were collected from university students in their real-life settings during 14 consecutive days with the help of smartphones.

The study was approved by the Institutional Review Board of the Department of Psychology of the University of Basel (IRB No. 016-17-2). All participants gave written informed consent prior to participation.

### Participants

45 psychology students from the University of Basel participated in this study. Due to technical problems with the smartphones, datasets from four participants were excluded, resulting in a final sample size of N = 41 participants (33 female). The mean age was 21.6 (SD = 3.9, range = 19–44 years). The number of semesters studied varied between 2 and 8 (mean = 3.2, SD = 1.5). In our final sample, 48.7% of the students reported being in a relationship, while 51.2% stated that they were single. Furthermore, 12.2% reported living alone, 60.9% with their families and 26.8% in shared accommodation. Participants were recruited via advertisements at our Faculty and were awarded course credits as compensation for their participation. Additionally, they were invited to take part in a lottery for 10 vouchers worth 20 Swiss Francs each.

### Procedure

#### ESM

In order to ambulatory assess the frequency/intensity of daily laughter by ESM as well as the experience of stressful events and stress symptoms (measures see below), we developed an app with a questionnaire for Android and installed it on the following devices: Samsung Galaxy S5 mini and Samsung Galaxy A3. The app was designed so that during a period of two weeks, the participants received prompts in form of an acoustic signal. The prompts occurred at randomized time intervals eight times per day, and in the ESM-sessions following each prompt the participants were asked to answer questions regarding the frequency/intensity of laughter as well as the stressful events and stress symptoms experienced since the last prompt. The prompts were delivered daily between 8.00 a.m. and 9.30 p.m. The minimum time interval between two prompts was set at 30 minutes. Participants were given the choice to activate a delay option twice per day to postpone filling out the survey in case they were unable to do so at the specific moment of receiving a prompt. If they did not answer the questions within 15 minutes after receiving a prompt, the survey for this particular prompt was automatically closed.

#### ESM-session

Each session began with two introductory questions (“Where are you?” and “Are you alone?”). These items only served as starter questions for the participants and were not analyzed. Subsequently, the participants were asked about the frequency/intensity of their laughter as well as the experience of stressful events and stress symptoms. These questions were arranged in three blocks presented in randomized order.

The study procedure was the following. Participants first took part in a group session to familiarize themselves with the study procedure. They were each provided with a smartphone with the newly developed app installed on it and shown how the app worked. They also filled out a demographic questionnaire. During the subsequent two weeks, they filled out independently the relevant questionnaires provided by the app after each acoustic prompt. At the end of the study period, they returned the devices to us. Finally, the participants were thanked, awarded the course credits earned, and informed about the purpose of the study.

### Measures

Laughter was assessed with a slightly revised version of the Daily Laughter Record [20], which we translated into German. We measured the frequency (“How often did you laugh since the last prompt?” with 0 = “not at all” to 5 = “very often”) as well as the intensity of laughter (“In the situations in which you laughed, how strong was your laugh?” with 1 = “weak” to 3 = “strong”) and the reason for laughing (“What was the reason that made you laugh?”; here, multiple answers were possible, e.g., “interaction with others”).

The experience of stressful events was assessed with the subscale “acute pressure of stress” of the Stress and Coping Inventory (SCI; [24]). Participants were asked whether they had experienced a stressful event since the last prompt (response options were “yes” or “no”).

The experience of stress symptoms was measured with a revised version of the subscale “physical and mental stress symptoms” of the SCI [24] consisting of, ultimately, eight self-rating items that required to be answered on a 6-point Likert-scale (“I suffered from stomach pressure or a stomach ache”, “I had a lump in my throat”, “I had a headache”, “I had twitchings/convulsions in my face that I could not control”, “I ruminated”, “I felt desperate”, “I was nervous”, “I felt restless”). We omitted four items that were not considered to be representative of daily fluctuations (e.g., sleep problems) and only included items which could plausibly be seen as being subject to variation within a short period of time. Satow [24] reported a Cronbach’s alpha of .86 for this scale, which can be regarded as very good. Using the nine SCI items, we constructed two outcomes of self-reported stress symptoms. First, we computed the average of items one through eight (specifying physical, cognitive, or emotional stress symptoms). However, one of the eight items (stress-related mimic twitches) was excluded from this averaged outcome on account of it being disconfirmed in most instances (item scale 0 through 5, median = 0). Second, we used the item “Since the last prompt, how much stress have you experienced” as a standalone outcome.

### Statistical analyses

We used linear mixed modeling (LMM; e.g., [25]) to answer our research questions. This type of modeling is suitable for analyzing hierarchical EMA data in which multiple observations are nested within subjects, with the number and timing of observations varying between subjects, and in which some observations are usually missing [26]. In both hypotheses, the outcome was the experience of stress symptoms, and the predictor was the experience of stressful events. In hypothesis 1, the respective moderator was reported frequency of laughter, while in hypothesis 2, the moderator was reported intensity of laughter.

All multilevel models included fixed and random effects. Fixed effects of the models were theoretically derived, while random effects were derived empirically, using best model-fit as the criterion. All models included in both parts of the mixed model the predictor, the outcome, the respective moderator variable, and, as potential confounding variables, time (EMA assessments) and sex, with the latter only featuring in the fixed effects part of the model. The random effects part of the model was kept at its empirically justifiable maximum, as recommended by [27]. Sex was included as a potential confounding variable since prior studies observed differences between men and women with regard to the frequency of daily laughter (e.g., [20, 21]).

In our analyses, the predictor and moderator were person-mean-centered, i.e., the zero value of a specific scale represents the individual’s mean score on that particular scale [25]. Prior to analysis, the outcome was log-transformed due to its right skewness, to better meet the LMM test assumptions. Finally, both the predictor and the moderator were lagged, so that we estimated the prospective association between the stressful event experienced at time T and the stress symptoms experienced at time T+1, moderated by either frequency or intensity of laughter at time T. Thus, a negative regression coefficient for the moderation term (as hypothesized by us) would mean that subjects who reported laughing more often (at T; frequency or intensity) also reported a subsequent lower level of stress symptoms (at T+1), compared to those reporting laughing less, always relative to the individual zero value of laughter. To examine the moderator effect of frequency and intensity of laughter respectively, we tested the cross-variable product between predictor and each of these two moderators.

As results we report fixed effects and present both unstandardized (usCOEF) and standardized model coefficients (sCOEF), each including their 95% confidence intervals (CI). Standardization was obtained by multiplying the regression coefficient by its standard deviation, divided by the standard deviation of the outcome [28].

All analyses were conducted in R (version 3.5.1) using the following packages: esmprep [29] to prepare the raw EMA data to be analyzed, nlme [30] for model estimation, effects [31] to compute the slopes for two levels of the moderator, and both cowplot [32] and ggplot2 [33] to visualize the moderation, and psych [34] to standardize model coefficients.

## Results

### Descriptive results pertaining to the EMA data

Overall, of 5008 prompted questionnaires, 94.6% were answered (median 96.7%), with a minimum of 75.6% and a maximum of 100%. The number of days during which EMA questionnaires were answered ranged from between 15 (seven participants) and 17 (one participant). The mean (as well as median) time that elapsed between successive prompts within the course of individual days was 102 minutes, mainly ranging from between 30 and 180 minutes (96.6% of all prompts). Less than 30 minutes or more than 180 minutes passed in 1.4% and 2%, respectively. There were direct cross-sectional effects of self-reported stressful events on stress symptoms. Using the combined measure of experienced stress symptoms as outcome, the LMM yielded an unstandardized association coefficient of 0.404, with a 95% confidence interval (95% CI) 0.350–0.457 (standardized: 0.374, 95% CI 0.324–0.432). Using the global (single item) measure of experienced stress symptoms as outcome, the unstandardized results were 0.669, with a 95% confidence interval (95% CI) 0.595–0.742 (standardized: 0.418, 95% CI 0.372–0.464). In both analyses we adjusted for sex.

### Hypothesis 1: Frequency of laughter attenuates the association between prior experience of stressful events and subsequent experience of stress symptoms in daily life

**Fig 1:**
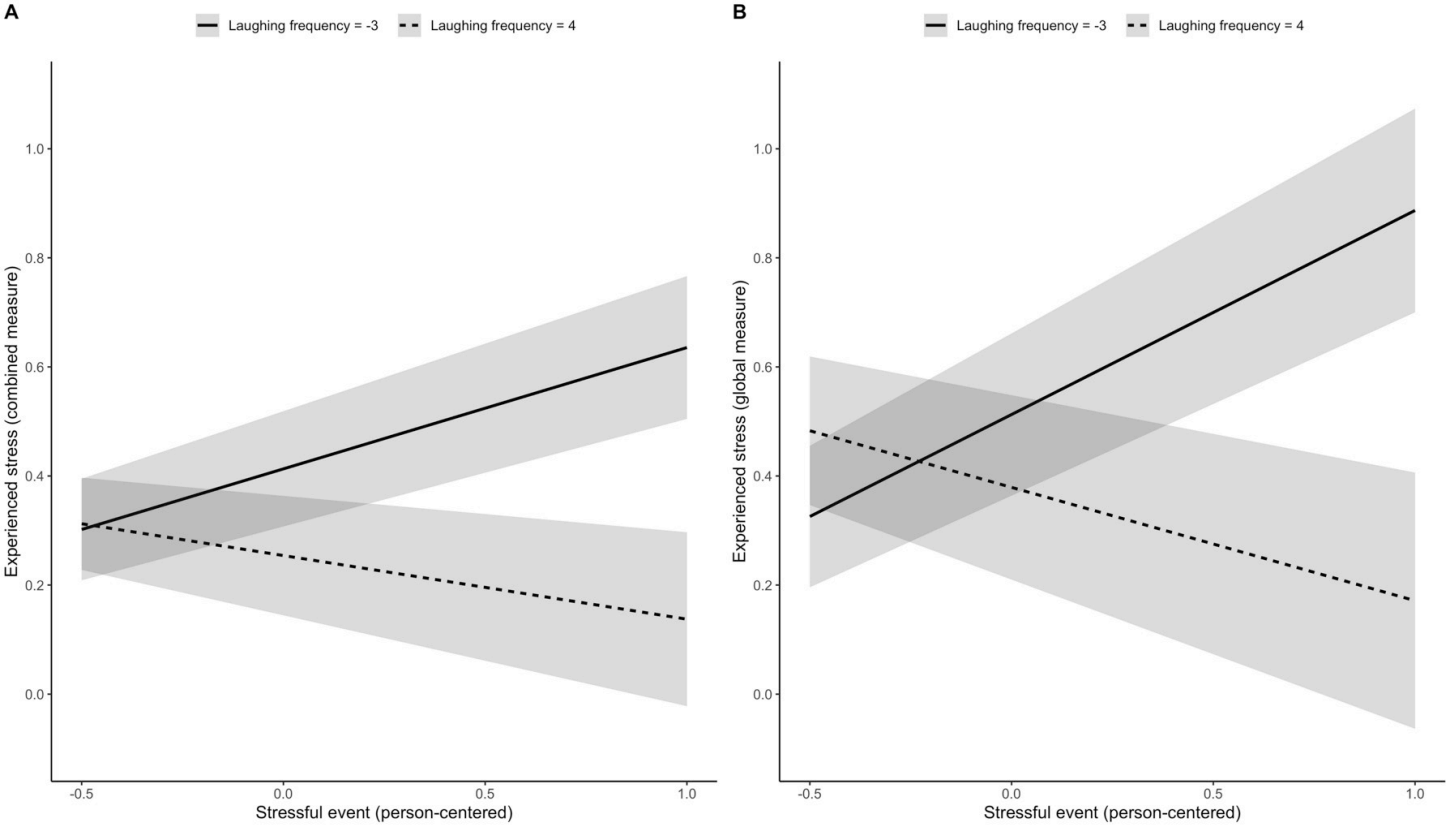
Laughing frequency moderating experienced stress. Moderator effect (with 95% confidence interval) of extreme values for frequency of laughter (person-centered) regarding the prospective association between stressful event experienced at time T and the stress symptoms experienced (aggregated self-report in plot A, global self-report in plot B) at time T+1. Compare results in Tables 1 and 2, respectively.

Using the combined measure of experienced stress symptoms, frequency of laughter moderated the association between experience of stressful events and subsequent experience of stress symptoms (see Table 1). Thus, the association between a previously experienced stress event and the subsequent experience of stress symptoms decreases by 0.048 (95% CI: -0.077 to -0.020; standardizied coefficient: -0.052; 95% CI = -0.082 to -0.021) when frequency of laughter increases by one standard deviation. Fig 1 illustrates the interaction effect for different levels of frequency of laughter. Using the global measure of experienced stress symptoms, the interaction effect remained stable (unstandardized coefficient: -0.083; 95% CI: -0.128 to -0.039; standardized coefficient: - 0.060: 95% CI: -0.092 to -0.028; see Table 2 and Fig 1).

**Table 1:** Results hypothesis 1: Frequency of laughter as moderator of the association between experience of stressful events and experience of stress symptoms (combined measure).

	Experience of stress symptoms (combined measure)					
Model variables	usCOEF	95% CI		sCOEF	95% CI	
(Intercept)	0.339	0.268	0.409			
Experience of stressful events	0.077	0.017	0.137	0.066	0.015	0.118
Frequency of laughter	-0.023	-0.033	-0.013	-0.085	-0.123	-0.047
Consecutive ema prompts	0.000	-0.001	0.001	0.011	-0.056	0.077
Sex (referent = female)	-0.002	-0.149	0.145	-0.002	-0.166	0.162
IA Experience of stressful events*Frequency of laughter	**-0.048**	-0.077	-0.020	**-0.052**	-0.082	-0.021

usCOEF: unstandardized model coefficient, sCOEF: standardized model coefficient; 95%CI: 95% Confidence Interval. IA: Interaction.

**Table 2:** Results hypothesis 1: Frequency of laughter as moderator of the association between experience of stressful events and experience of stress symptoms (global measure).

	Experience of stress symptoms (global measure)					
Model variables	usCOEF	95% CI		sCOEF	95% CI	
(Intercept)	0.477	0.387	0.568			
Experience of stressful events	0.125	0.053	0.197	0.072	0.031	0.114
Frequency of laughter	-0.019	-0.037	-0.001	-0.048	-0.093	-0.003
Consecutive ema prompts	0.000	-0.001	0.001	-0.012	-0.074	0.051
Sex (referent = female)	-0.069	-0.270	0.132	-0.052	-0.203	0.099
IA Experience of stressful events* Frequency of laughter	**-0.083**	-0.128	-0.039	**-0.060**	-0.092	-0.028

usCOEF: unstandardized model coefficient, sCOEF: standardized model coefficient; 95%CI: 95% Confidence Interval. IA: Interaction.

### Hypothesis 2: Intensity of laughter attenuates the association between prior experience of stressful events and subsequent experience of stress symptoms in daily life

Evaluating intensity of laughter as moderator, the interaction effects for both outcome measures contained the zero value in the 95% CI (see Tables 3 and 4). Therefore, hypothesis 2 was not confirmed.

**Table 3:** Results hypothesis 2: Intensity of laughter as moderator of the association between experience of stressful events and experience of stress symptoms (combined measure).

	Experience of stress symptoms (combined measure)					
Model variables	usCOEF	95% CI		sCOEF	95% CI	
(Intercept)	0.347	0.274	0.420			
Experience of stressful events	0.096	0.038	0.153	0.082	0.033	0.131
Intensity of laughter	-0.020	-0.039	-0.002	-0.034	-0.065	-0.003
Consecutive ema prompts	0.000	-0.001	0.001	-0.008	-0.076	0.061
Sex (referent = female)	-0.023	-0.184	0.138	-0.026	-0.205	0.154
IA Experience of stressful events* Intensity of laughter	0.026	-0.042	0.093	0.012	-0.020	0.045

usCOEF: unstandardized model coefficient, sCOEF: standardized model coefficient; 95%CI: 95% Confidence Interval. IA: Interaction.

**Table 4:** Results hypothesis 2: Intensity of laughter as moderator of the association between experience of stressful events and experience of stress symptoms (global measure).

	Experience of stress symptoms (global measure)					
Model variables	usCOEF	95% CI		sCOEF	95% CI	
(Intercept)	0.493	0.399	0.587			
Experience of stressful events	0.128	0.065	0.192	0.075	0.038	0.111
Intensity of laughter	-0.007	-0.036	0.022	-0.008	-0.041	0.025
Consecutive ema prompts	0.000	-0.001	0.000	-0.030	-0.090	0.030
Sex (referent = female)	-0.045	-0.262	0.171	-0.034	-0.197	0.129
IA Experience of stressful events* Intensity of laughter	0.100	-0.006	0.205	0.032	-0.002	0.066

usCOEF: unstandardized model coefficient, sCOEF: standardized model coefficient; 95%CI: 95% Confidence Interval. IA: Interaction.

## Discussion

This study investigated in a student sample whether the association between experiencing a stressful event and the subsequent experience of stress symptoms was attenuated by frequency and intensity of laughter. As expected, we found that the association between stressful events and subsequent stress symptoms was moderated by the frequency of laughter experienced at the time of the stressful event (H1). The more frequently students laughed proximal to experiencing stressful events, the weaker the association between stressful events and stress symptoms became. With students’ frequency of laughter increasing (relative to an individual’s mean frequency), the prospective association between experiences of stressful events and self-reported stress symptoms decreased. When individuals laughed often, stressful events were even associated with lower stress symptoms. The moderating effect of frequency of laugther was found for global stress measures as well as for composite measures of stress symptoms, which strengthens our confidence in the findings of this study. Thus, our results support the notion that the stress-buffering effect of positive affect [8] also applies to situations in which stress and positive affect occur in close temporal approximation. As our sample was predominantly female, this conclusion applies primarily to women, and future studies are invited to investigate more closely potential gender-related differences in the stress-buffering effects of positive affect. Nevertheless, our results extend previous findings from laboratory studies and longitudinal studies to daily life situations in a student population, using ambulatory assessments.

Surprisingly, however, intensity of laughter was found to have no moderating effect on the association between experiencing a stressful event and subsequent stress symptoms, and our hypothesis 2 was therefore not confirmed. Several factors may explain the discrepancy in findings between frequency and intensity of laughter. For example, it is possible that the self-report of intensity of laughter is less reliable than the self-report of frequency of laughter. In particular, people may have difficulties in specifically recalling differing degrees of intensity of laughter, whereas frequency of laughter may be easier to quantify (see also limitations below). Alternatively, intensity of laughter might simply be a less reliable indicator of positive affect than frequency of laughter. Future studies should therefore disentangle the potentially distinct relationships between stress alleviation and frequency and intensity of laughter, respectively.

The strengths of our study lie in the following. First, we chose an intensive longitudinal study design with up to 123 measurements per participant (i.e., 8 questionnaires per day over a period of 14 consecutive days). Second, on average 94.6% of all questionnaire prompts were answered by our 41 participants, yielding a relatively small rate only of missing observations. Third, we report prospective associations (by establishing a temporal order of prior exposure and subsequent outcome), an essential criterion when discussing potential causality [35, 36]. Fourth, we investigated the stress-buffering effect of positive affect outside the laboratory, thereby enhancing ecological validity and being able to infer real-life dynamics.

Despite these strengths, this study faces the following potential limitations and opens up various avenues of future investigation: First, our study is an observational study, not an experimental one. This design does not allow for direct causal implications to be inferred. Second, our assessment does not allow the temporal disentanglement of stress from laughter. As a starting point, future studies are invited to consider different time intervals when modeling possible stress-buffering effects of positive affect on subsequent outcomes. Given that certain stress symptoms might take some time to develop, it would be interesting to test other forms of lagged models. Third, no physiological stress measures were applied. Participants in our study completed only self-evaluation measures. Individuals, however, may differ in their abilities to self-report the intensity and frequency of laughter, and individuals’ self-reports may be prone to biases (e.g., including mood effects). Future studies might consider the use of devices such as the Electronically Activated Recorder (EAR) to detect the intensity and frequency of laughter more objectively. Previous studies were already successful in applying the EAR as a method to investigate daily social behavior [37, 38]. Future studies are also invited to include physiological stress measures, such as cortisol levels or puls rates. Fourth, our sample consisted of a fairly homogenous group of young, predominantly female students from the University of Basel, which is why our results are not generalizable to the general population. A last limitation lies in the fact that only few stressful events (ca. 12%) were reported by the students, and we thus cannot ascertain with any certainty whether the observed moderation effect pertains to populations experiencing more stressful events. For instance, the stress-buffering function of laughing in the context of experiencing one single, only mildly stressful event might be different from that of laughing in the context of multiple, highly stressful events. However, results of a recent study investigating the prospective association between positive affect and longevity suggest that the link between positive affect and positive health outcomes is more pronounced in people who experience high stress levels [17]. Thus, it would be interesting to explore whether this holds also true in daily life, where the experience of different affect and stress levels are subject to frequent rapid change. Extending this, another future research perspective may be to investigate whether certain kinds of traits may have an influence on the stress-buffering effect of positive affect. For instance, it might be interesting to explore whether the stress-buffering effect of positive affect is stronger in individuals with high trait mindfulness and/or high emotion regulation skills as both are per se associated with greater levels of positive affect and well-being [39, 40, 41].

To conclude, our study adds important empirical evidence to the stress-buffering model of positive affect [8]. Future studies need to replicate our findings in order to test the model’s robustness. A future EMA design might include wearables with reliable sensor technique to assess physiological symptoms of stress at the moment of occurrence such as pulse rate or skin conductance level, possibly in populations experiencing high levels of stress in their daily lives.
